# Synthesis of crystalline and amorphous, particle-agglomerated 3-D nanostructures of Al and Si oxides by femtosecond laser and the prediction of these particle sizes

**DOI:** 10.1186/1556-276X-7-619

**Published:** 2012-11-09

**Authors:** Mugunthan Sivayoganathan, Bo Tan, Krishnan Venkatakrishnan

**Affiliations:** 1 Department of Aerospace Engineering, Ryerson University, 350 Victoria Street, Toronto, Ontario, M5B 2K3, Canada; 2 Department of Mechanical and Industrial Engineering, Ryerson University, 350 Victoria Street, Toronto, Ontario, M5B 2K3, Canada

**Keywords:** 3-D nanostructures, Synthesis, Al and Si oxides, Crystalline, Amorphous, Characterization, Prediction, Agglomerated particle size, Plume properties, 81 Materials science, 81.07.-b nanoscale materials and structures: fabrication and characterization, 81.16.-c methods of micro- and nanofabrication and processing

## Abstract

We report a single step technique of synthesizing particle-agglomerated, amorphous 3-D nanostructures of Al and Si oxides on powder-fused aluminosilicate ceramic plates and a simple novel method of wafer-foil ablation to fabricate crystalline nanostructures of Al and Si oxides at ambient conditions. We also propose a particle size prediction mechanism to regulate the size of vapor-condensed agglomerated nanoparticles in these structures. Size characterization studies performed on the agglomerated nanoparticles of fabricated 3-D structures showed that the size distributions vary with the fluence-to-threshold ratio. The variation in laser parameters leads to varying plume temperature, pressure, amount of supersaturation, nucleation rate, and the growth rate of particles in the plume. The novel wafer-foil ablation technique could promote the possibilities of fabricating oxide nanostructures with varying Al/Si ratio, and the crystallinity of these structures enhances possible applications. The fabricated nanostructures of Al and Si oxides could have great potentials to be used in the fabrication of low power-consuming complementary metal-oxide-semiconductor circuits and in Mn catalysts to enhance the efficiency of oxidation on ethylbenzene to acetophenone in the super-critical carbon dioxide.

## Background

Ceramic nanostructures have attracted much attention due to their extended applications in science and technology. They can be used in many modern applications, such as biomedical, electronics, photonics, hot gas filtration, gas adsorption and desorption (hydrogen gas for fuel cell technology), and in thermophotovoltaics. Several different techniques are used for the synthesis of ceramic nanostructures. These techniques include sol–gel [1,2], chemical synthesis [3], porous-anodic alumina [4], and electrospinning [5].

The recent research on ceramics has shown the formation of nanoparticles by laser ablation in aqueous solution [6]. Laser ablation has been rarely used for the synthesis of ceramic nanostructures in standard atmospheric conditions. It could be due to the heat accumulation owing to poor thermal diffusivity of material, which leads to an increase in brittleness by laser-induced thermal stresses. This results in the formation of irregular chips during ablation. In this study we report a single step technique to synthesize free-standing, particle-agglomerated, amorphous 3-D nanostructures of Al and Si oxides from powder-fused aluminosilicate ceramic and a simple novel method to fabricate crystalline nanostructures of Al and Si oxides with the same morphology using femtosecond laser at atmospheric conditions.

Aluminosilicate ceramic is a biomedical material that has shown biocompatibility in simulated body fluid. It is applied in bone filling, protein absorption, and nanobiocomposites [7-10]. The sol–gel dry spinning [11] technique has been extensively used for the fabrication of highly pure, homogeneous, crack-free aluminosilicate ceramic microfibers at low temperature. However, no studies showed the synthesis of 3-D particle-agglomerated nanostructures on aluminosilicate ceramic by laser ablation.

Studies show that the mixed oxide structures generated in our lab can have great potential in several applications. For instance, a very recent study showed the fabrication of low power-consuming complementary metal-oxide-semiconductor (CMOS) circuits with buried thin layers of Al_2_O_3_ and SiO_2_ on bonded GeOI substrates [12]. Another study showed that the presence of mixed nanosized SiO_2_ and Al_2_O_3_ increases the efficiency of Mn catalysts for the oxidation of ethylbenzene to acetophenone in super-critical carbon dioxide [13]. Our technique shows an easy way to produce mixed oxide nanostructures and control their ratio by varying the laser operating parameters. In addition, it opens the possibility of generating both amorphous and crystalline structures which could be used to further enhance possible applications [14-16].

In mixed oxide nanostructures, not only the mixing ratio of compositions play a key role in controlling their properties, but also their size. To analyze the effects of laser parameters on the size of generated nanostructures, a study was performed on the size distribution of agglomerated particles in these structures. Few previous studies were performed to analyze the effect of laser parameters, such as laser fluence and laser intensity, on the average size and size dispersion of singly distributed nanoparticles [17,18]. However, no studies were performed on agglomerated particles of laser-generated nanostructures, and no mechanism was suggested to predict the particle size distributions.In this article, we show two different simple laser ablation techniques to fabricate the amorphous and crystalline nanostructures of Al and Si oxides and propose a mechanism to predict the size of agglomerated particles with laser parameters, plume properties, and nucleation rate. This mechanism could help to regulate the size of agglomerated nanoparticles in these structures and to modify the properties of the fabricated nanostructures. The predicted mechanism is not necessarily restricted to the selected material or to this particular study.

## Methods

A 1,030-nm wavelength, directly diode-pumped, Yb-doped, fiber-amplified femtosecond laser system was used in performing the experiments. The laser delivered a maximum output power of 11.0 W (power at the material surface) with a repetition rate ranging from 200 kHz to 25.2 MHz and a pulse duration of 200 fs. During the experiment, pulse duration and laser spot size were kept constant. Experiments were performed at the repetition rate of 25.2 MHz with varying dwell times (time spent delivering laser pulses to a single point on the sample). A galvoscanner was used to irradiate the samples on a predetermined laser scan pattern. The scan pattern was an array of points with a center-to-center distance of 100 μm. The spot size of the beam at the sample was kept at 10.38 μm. All samples were ablated under the ambient conditions. The first set of experiments was performed on powder-fused aluminosilicate ceramic thin plates with a chemical composition of 29.2% (by weight) Al_2_O_3_, 59% SiO_2_, and trace elements. Initially, threshold fluence of ceramic aluminosilicate plate at 25.2 MHz was found. The dwell time was kept at 0.1 ms. The threshold fluence was obtained by increasing the laser power until the initial point of ablation obtained. The results were confirmed with decreasing power also. Then different sets of samples were processed with varying fluence to threshold ratios (*fluence ratio*) by simply varying the laser power. In our studies, the ratio between fluence and threshold fluence was maintained as a comparison bench mark. The second set of experiments were performed on a combined Al foil-Si wafer set up with a power of 11 W (power at sample) and a repetition rate of 25.2 MHz with varying dwell times: 3, 5, and 9 ms. The generated nanostructures on both sets of experiments were studied using a scanning electron microscope (SEM) and a transmission electron microscope (TEM).

## Results and discussion

### Synthesis of amorphous nanostructures of Al and Si oxides from powder-fused aluminosilicate plates

**Figure 1:**
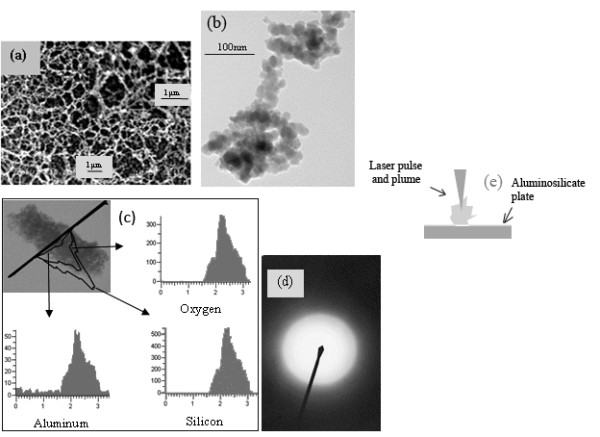
**Structure characterization and laser processing.** (**a**) SEM images (the insert at the right top is a close-up), (**b**) TEM image, (**c**) TEM-EDX analysis, (**d**) TEM diffraction analysis of 3-D nanostructure formed around the laser irradiated spot during the femtosecond laser ablation of aluminosilicate ceramic at 11.0 W and 25.2 MHz, and (**e**) ablation process of aluminosilicate ceramic.

Femtosecond laser ablation is used to synthesize nanostructures of Al and Si oxides on powder fused ceramic aluminosilicate plates. Figure 1a shows the SEM image of the fabricated 3-D nanostructure, which is formed in an array of irregular ring shapes. TEM (Figure 1b) of fabricated nanostructures shows an agglomerated morphology of nanoparticles. The TEM-energy dispersive X-ray spectroscopy (TEM-EDX) micrograph in Figure 1c shows the analysis of elements in the generated nanostructure. Further, TEM diffraction analysis performed on the nanostructures (Figure 1d) confirms the amorphous nature of these structures. The laser ablation set up is shown in Figure 1e.

The powder-fused aluminosilicate used in our experiments has a chemical composition of 29.2% (by weight) Al_2_O_3_, 59% SiO_2_, and trace of other elements. Calculations on the chemical composition showed that the unablated sample contains silicon and aluminum in the ratio of 5:3, but the TEM-EDX analysis of formed 3-D nanostructure (Figure 1c) has a ratio of 10:1 for the same elements. This shows that a higher amount of silicon was ablated than aluminum from the aluminosilicate ceramic substrate in the form of element or compound. One of the possible reasons for the variation in elements ratio for unablated and ablated samples is that a higher number of Si-O bonds could have been broken than Al-O bonds during ablation from the parent material. This could happen if Al-O bond is stronger than Si-O bond. Another possible reason for the variation of these above elements in nanostructure could be that the difference in the rate of evaporation of compounds such as SiO_2_ and Al_2_O_3_. These results could be explained with Pauling's electronegativity concept which relates the bond strength to its percentage of covalency and ionicity. An increase in covalency of a bond decreases its ionicity and increases its bond strength. Previous studies have shown that within an aluminosilicate ceramic, the Al-O bonds are more covalent and stronger than Si-O bonds and also the melting point of pure Al_2_O_3_ is higher than pure SiO_2_[19]. These studies agree well with the above presented results. The above detailed method produced amorphous nanostructures of Al and Si oxides. A similar method with a different set up is proposed to synthesize crystalline nanostructures of Al and Si oxides.

### Synthesis of crystalline nanostructures of Al and Si oxides by combined silicon wafer-aluminum foil ablation

**Figure 2:**
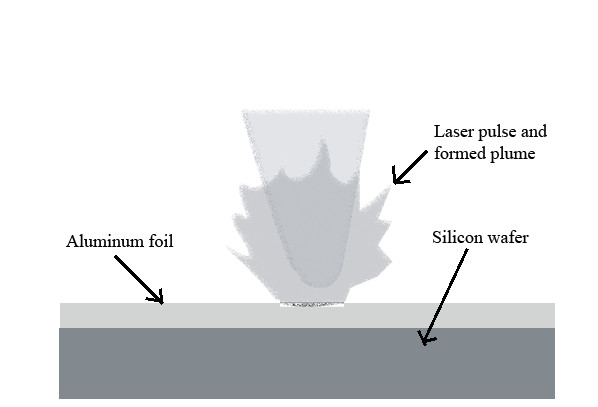
Arrangement of combined silicon wafer-aluminum foil ablation for synthesis of Al-Si oxides nanostructures.

A simple novel technique was used to fabricate crystalline nanostructures of Al and Si oxides by Al foil-Si wafer-combined ablation. A piece of aluminum foil with a thickness of 10 μm was fixed on top of an undoped <100>−oriented 99.96% pure silicon wafer. The compound was ablated by a 25.2-MHz, 11.0-W (power at sample surface) femtosecond laser for three different sets of dwell times - 3, 6, and 9 ms. Figure 2 shows the experimental set up used for the novel synthesis.

**Figure 3:**
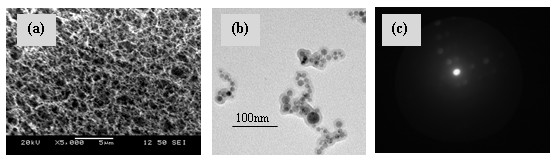
**Material characterization studies on the generated nanostructures.** (**a**) SEM image, (**b**) TEM image, and (**c**) crystallinity test image obtained on Al-Si nanooxides for the dwell time of 5 ms ablation.

Figure 3a,b shows SEM and TEM images obtained on the generated structures. The TEM diffraction study performed on the fabricated nanostructures shows the crystalline nature of the substances. Figure 3c shows one of the TEM diffraction image obtained on ablated sample at 5 ms dwell time.

Table 1 shows the TEM-EDX analysis for three different sets of experiments performed. The variation of Al/Si ratio shows the effect of progressive ablation of the Al foil-Si wafer with an increase in dwell time. This technique could be used to vary the composition of fabricated Al-Si nanooxides by just varying the laser parameters inclusive of dwell time as well as the thickness of the aluminum foil. The properties of the mixed oxide nanostructures could be varied by varying the ratio of distribution of both oxides as well as the size of the fabricated nanostructure. To relate the size of the fabricated nanostructure to the laser parameters and to regulate the size of these structures, a particle size distribution study was performed on the fabricated agglomerated particles of powder-diffused aluminosilicate ceramic. A mechanism is also suggested to predict the size of agglomerated particles of these generated structures.

**Table 1:** TEM-EDX of crystalline nanostructures of Al and Si oxides for varying dwell times

**Element**	**Dwell time**		
	**3 ms**	**5 ms**	**9 ms**
Aluminum	30.06	18.99	3.81
Silicon	9.58	15.67	49.77
Oxygen	60.36	65.33	46.42

### Prediction of agglomerated particle sizes in 3-D amorphous nanostructures of Al and Si oxides

The knowledge of different particle-forming mechanisms is vital to analyze the results obtained in our experiments. Basically, the particle-generating mechanisms could be divided into two major categories: vapor condensation and melt ejection. In most of the laser ablating conditions, a combination of both vapor condensation and melt ejection is involved.

During nucleation, the vapor plume condenses and forms nuclei which further grow into nanoparticles. Formation of the nucleus is governed by the temperature and concentration of the nucleating species (atoms, molecules, and clusters) and the properties of the material being ablated [20]. Further, previous studies have shown that the laser-ablated plume temperature increased with increasing laser fluence and the size of the clusters formed by the vapor phase nucleation are in the nanometer range [21-23].

Other particle-forming mechanisms involved during laser ablation are hydrodynamic sputtering, spallation, phase explosion, and solid exfoliation. Other than solid exfoliation, all of the above given mechanisms involve particle formation by liquid melt ejection. Solid exfoliation involves irregular solid state particle removal due to laser-induced thermal stresses. Further, previous studies showed that the sizes of particles formed by all of the above given melt ejection mechanisms were greater than 100 nm (i.e., in microscale). There could be a few other mechanisms that exist during laser ablation, such as fragmentation and coalescence. These mechanisms do not directly form particles, but change the size of already formed particles.

**Figure 4:**
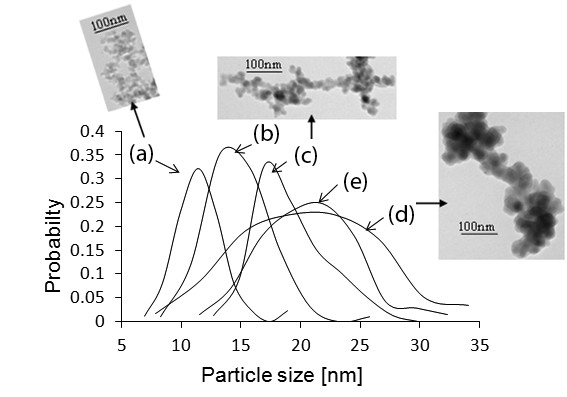
**Particle size distribution curves for varying fluence ratios.** (**a**) 1.6, (**b**) 2.0, (**c**) 2.4, (**d**) 2.8, and (**e**) 3.2 for constant repetition rate of 25.2 MHz. The sample of TEM images obtained at (**a**) 1.6, (**c**) 2.4, and (**d**) 2.8 fluence ratios.

**Figure 5:**
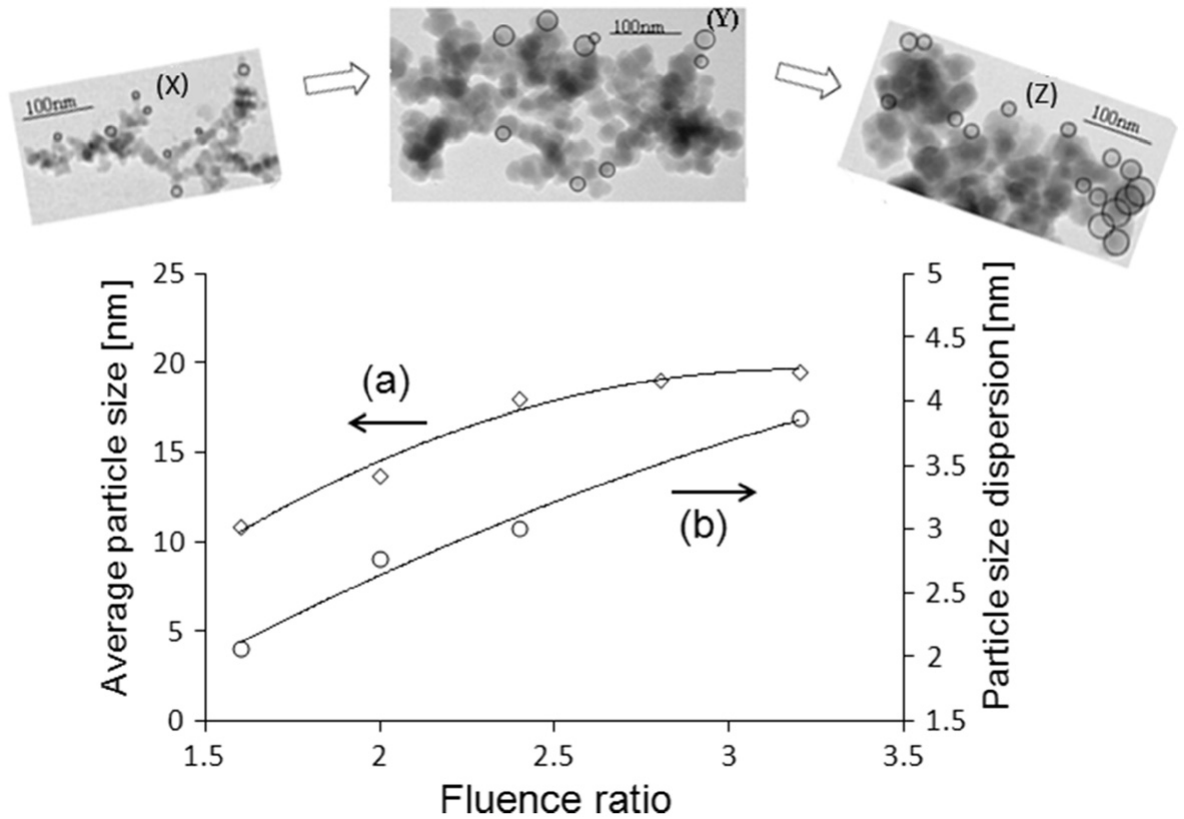
**Particle size characterization.** (**a**) Average particle sizes and (**b**) particle size dispersions which are obtained in the 3-D nanostructures for different fluence ratio at constant repetition rate of 25.2 MHz. The sample TEM images with increasing fluence ratio is shown along (X-Y-Z).

To form a prediction mechanism of particles in agglomerated nanostructures, the TEM images of 3-D nanostructures obtained during our experiments were analyzed. Figure 4 shows that the structures were made out of agglomerated particles. The spherical nanoparticles are agglomerated to form three-dimensional nanostructures. The particle size characterization studies showed that the range of particles in the three-dimensional nanostructure is from 7 to 31 nm. This range indicates that the particles in our structures are made by vapor condensation [21]. The particle size distributions in 3-D nanostructures (shown as (a-e) in Figure 4) and their averages and the dispersions (shown as (a and b) in Figure 5) were found.

A mathematical modal study of laser-induced particle formation showed that the nanoparticle sizes formed by vapor condensation fit within a bimodal distribution curve [20]. Our results, however, showed unimodal distribution rather than bimodal distribution. A possible reason could be the difference in the ablated material in our studies and the presence of species in the formed plume.

TEM images in Figures 4 and 5 show an increase in the average particle size and their dispersion with increasing fluence ratio. The size of the 3-D structure could vary with the size of the agglomerated particles. Our study shows that the laser fluence played an important role in determining the size of the agglomerated particles in the nanostructures and thus the size of the structures. The average particle sizes in 3-D nanostructures increased from 10 to 20 nm, when the fluence ratio was increased from 1.6 to 2.4. The average particle size remained at an approximately constant diameter of 20 nm, when the fluence ratio was increased beyond 2.4 to 3.2. The corresponding changes in laser intensities and laser fluences are 3 × 10^10^ to 6 × 10^10^ W/cm^2^ and 0.37 to 0.84 J/cm^2^.

The results obtained for agglomerated particles in the nanostructures have a good correlation with those obtained previously by different researchers for the size variation of singly distributed, non-agglomerated particles. Vitiello et al. [17] have shown the same trends in the averages and the dispersions of gold nanoparticle sizes produced using laser fluence and laser intensity values from 0.3 to 1.1 J/cm^2^ and 3 × 10^12^ to 1.1 × 10^13^ W/cm^2^, respectively. Another study of fabricated nickel and silicon nanoparticles showed similar trends in the range of particle sizes with laser intensity [18]. Though the results show the same trends in distribution, our study totally differs from the rest as the particles are studied in agglomerated state which determines the size of the 3-D nanostructures as well as their properties.

Laser-material interaction studies have shown that the laser plume temperature increases with the increase in laser energy and intensity [22,23]; also, the amount of supersaturation and the nucleation rate decrease with the increase in the plume temperature [20,24-26]. Vapor condensation theories showed the rate of nucleation increases faster than the growth rate of particles with the increase in amount of supersaturation. Hence, the average sizes of formed particles decrease with an increase in supersaturation [27]. By combining all those previous research results, we formed a mechanism to predict the particle size as well as our experimental results. The variation in average particle size in Figure 5 could be explained as follows: increasing (plume) temperature leads to a chain of effects. There is an increase in (plume) pressure, a decrease in the amount of supersaturation, and a decrease in the nucleation rate as well as the growth rate. Though both the nucleation rate and the growth rate decrease with increase in plume temperature, the effect of the decrease in nucleation rate overcomes the growth rate, which results in an increase of the average particle size formed [24-29].

At very high fluence ratios (between 2.4 and 3.2), the plume temperature saturation could be reflected also by the saturation (approximately) in the average particle size in Figure 5 (a) [30]. Though plume temperature could be saturated at high fluences, the material ablation could continuously take place. This could lead again to an increase in supersaturation of plume, where again the competition between the nucleation rate and particle growth rate could be occurred, which is feasible from (d and e) in Figure 4. A drop in the particle size distribution is obtained, when the laser fluence increased from 2.8 to 3.2

The range of particle size increases with fluence ratio because the nucleation of particles could be occurred until the final constant condensation temperature is reached [21]. Figure 5 (b) shows an increase in particle size dispersion from 2 to 4 nm with the increase in fluence ratio. Further, this may be due to the change in plume size and as well as the particle cooling rate at different parts of the plume with the increase in laser fluence.

The effect of changes in laser fluence along the used Gaussian laser pulse could be reflected by the particle size distribution obtained in (a-e) in Figure 4. Fewer large-sized particles could represent peak fluence, fewer small-sized particles could represent edges with low fluence, and more average size particles could represent average fluence portions of the laser profile. There is always a high possibility of more than one mechanism taking place during particle formation [31]. However, the particle size characterization studies confirmed the presence of only vapor-condensed particles. It could be due to a short time lapse between the melting and evaporation stages during femtosecond laser ablation.

Disagreements may exist in applying nucleation theory for nanoscaled structures, but the studies in aerosol particle formation performed previously by researchers confirmed the likelihood of this theory. Another study showed that nucleation theories could be applied, when the critical size is greater than 16 to 20 molecular formulas [32]. For different gas–liquid nucleation conditions, nucleation theories showed a consistent underestimation in the nucleation rates for different temperatures and supersaturations. However, the trends in changes of averages and dispersions of particle size presented agreed well with the nucleation theories [33].

## Conclusions

This study showed a single step technique to synthesize amorphous and crystalline three-dimensional nanostructures of Al and Si oxides at ambient conditions on aluminosilicate ceramic plates made from powder metrology and by a simple novel method of foil-wafer ablation. A mechanism was proposed to predict and regulate the size of agglomerated nanoparticles in these fabricated structures with laser parameters, plume properties, and nucleation rate. Variation in laser parameters also varies plume temperature, pressure, and amount of supersaturation, nucleation and growth rates of vapor-condensed nanoparticles, and the size of the formed nanoparticles. The suggested prediction mechanism is not necessarily restricted for this particular study or material. This prediction method could be used to produce 3-D nanostructures with unique particle size, which will enhance the properties of these structures and their performance in various applications. Further, the fabricated nanostructures of Al and Si oxides could have a great impact in the fabrication of low-power-consumption CMOS circuits and in increasing the efficiency of Mn catalysts for the oxidation of ethylbenzene to acetophenone in a super-critical carbon dioxide.

## Abbreviations

Al: Aluminum; EDX: Energy dispersive X-ray spectroscopy; SEM: Scanning electron microscope; Si: Silicon; TEM: Transmission electron microscope.

## Competing interests

The authors declare that they have no competing interests.

## Authors’ contributions

MS performed the laser ablation experiments, SEM, TEM and EDX measurements, and developed the proposed particle size prediction mechanism. KV proposed the idea of novel wafer-foil ablation. BT and KV supervised the analyses of materials, vetting of the proposed particle size prediction mechanism, and preparation of the document. All authors have read and approved the final manuscript.
